# Comparison of the Clinical Outcomes of Titanium and Zirconia Implant Abutments: A Systematic Review of Systematic Reviews

**DOI:** 10.3390/jcm11175052

**Published:** 2022-08-28

**Authors:** Felita Clarissa Halim, Paolo Pesce, Nicola De Angelis, Stefano Benedicenti, Maria Menini

**Affiliations:** 1 Private Practice, Jakarta 13220, Indonesia; 2 Division of Prosthodontics and Implant Prosthodontics, Department of Surgical Sciences (DISC), University of Genoa, 16132 Genoa, Italy; 3 Division of Restorative Dentistry and Endodontics, Department of Surgical Sciences (DISC), University of Genoa, 16126 Genoa, Italy; 4 Dental Department, University Tunku Abdul Raman, Sungai Buloh 47000, Malaysia; 5 Dental Department, University Trisakti, Jakarta 11440, Indonesia

**Keywords:** dental implants, abutment, zirconia, systematic review of systematic reviews

## Abstract

Background: Dental implants are widely used and in order to answer to esthetic demands, zirconia has been introduced as an abutment material as an alternative to titanium. Several studies have been published on this topic, but the results have been often inconsistent. The objective of the present study is to systematically analyze the existing literature comparing clinical outcomes of titanium and zirconia implant abutments. The study was designed as a systematic review of systematic reviews. Methods: This systematic review is in accordance with the Transparent Reporting of Systematic Reviews and Meta-analyses. A MEDLINE/PubMed, Cochrane Database of Systematic Reviews and SCOPUS literature search was performed up to and including June 2021. Data were extracted independently by two reviewers and tAMSTAR2 was used to assess the quality of the systematic reviews. Results: The electronic search identified 1146 papers, and 175 duplicates were removed. After manual screening, 954 studies were excluded and the final analysis was conducted on 11 papers. Both mechanical and esthetic outcomes and biological complications were analyzed. Conclusions: It can be concluded that titanium abutments have a better mechanical resistance than zirconia ones. Plaque accumulation is reported to be slightly higher on titanium but without any significant inflammatory process. The esthetic outcomes seem to be more related to the thickness (>3 mm) of the soft tissues than to the abutment material.

## 1. Introduction

Dental implants are often used to restore partially and completely edentulous patients due to their reported long-term survival and success [1]. Dental implants are considered survived when they are osseointegrated. The obtainment and maintenance of osseointegration is affected by several factors including the surface properties of titanium implants, which influence molecular interactions at the bone–implant interface, the cellular response and, finally, bone remodeling [2]. Long-term retrospective cohort studies have investigated the relationship between various factors that might influence the implant survival rate [3]. Note that survival means that the implants are still present in the patient’s mouth, independent of biologic and/or technical complications, and does not capture a successful treatment [3,4].

Titanium implant surface modifications have been evaluated over the past years and scientific studies have shown that rougher surfaces have a better and more rapid bone formation compared to machined titanium implant surfaces. Modifications of the implant surfaces are made with many different methods such as machining, air-abrasion, acid etching, electrochemical oxidation, and laser treatment. The surface roughness plays an important role for cellular reactions, tissue healing, and implant stability [2]. On the other hand, rough surfaces can accumulate subgingival plaque up to 25 times more than smooth or machined surfaces. This might promote plaque accumulation as well as the subsequent pathology of peri-implant tissues, although a direct correlation with bone resorption has not been demonstrated [5,6,7,8].

A successful osseointegration is defined as a direct bone-to-implant contact without the interposition of any other tissue, and in order to preserve osseointegration it is desirable to have no parafunctional forces, mal-aligned forces, peri-implantitis, an absence of systemic diseases, and to consider the host immune-inflammatory response to bacterial challenges [9]. Besides these, there are also triggering factors that may cause peri-implant bone loss and eventually implant failure that are didactically divided into two main categories: biological factors (for example, the presence of bacterial strains) and biomechanical factors (for example, excessive mechanical stress) [5,10].

It has been well documented in the literature that peri-implant bone supporting two-piece implants undergoes crestal bone loss after the placement of the abutment and delivery of the prosthesis in a single tooth replacement, and in both partially and completely edentulous patients. Among the many factors that cause bone loss, the early stages of bone loss are usually caused by an overload, a micro-gap, a polished implant neck and infection. While the reasons for early crestal bone loss have been extensively studied in the last decade, the stability of the crestal bone over time remains a controversial issue. The micro-gap at the implant–abutment interface [11], has been proven to be a possible triggering factor if placed at the bone level or below. Placing the implant–abutment interface supracrestally can avoid early crestal bone loss, but might represent an esthetic issue [12,13].

It must also be considered that peri-implant soft tissue serves as a protective seal between the oral environment and the underlying peri-implant bone [2,14], and its health is associated with a reduction in the risk of bone resorption [2]. Biological implant-associated complications usually begin in peri-implant soft tissue. Connective tissue and the epithelium of peri-implant soft tissue are in direct contact with the transmucosal implant abutments [15]. Collagen fibers surrounding an implant and a tooth are not the same. For example, natural teeth present perpendicular fibers inserted into the cementum, while collagen fibers around implants are mostly circular and parallel to the implant surface; hence, the peri-implant seal is considered weaker and can be easily invaded by prosthetic cement and contaminated by oral bacteria [16]. The characteristics of the implant prosthetic abutment are considered to be an influential factor which may impact early bone remodeling and soft tissue integration. The abutment material, as surface microtopography [1] has shown, influences the response of both the soft and hard tissues surrounding an implant, making the choice of the prosthetic abutment a crucial phase for the success of implant-supported rehabilitations [17].

Standardized titanium abutments represent the gold standard for implant reconstruction due to the good stability reported in several clinical studies [18]. For many years, the standard stock abutments provided by implant manufacturers were the only option available for clinicians [17]. These prefabricated components may simplify technical procedures; however, they also exhibit several shortcomings, especially in the esthetic aspect. Firstly, the natural emergence profile of a reconstruction cannot be achieved with prefabricated abutments because of the cylindrical cross-section. In order to achieve a natural emergence profile, modifications can be made in the shape of the crown which often leads to over contouring of the reconstruction. Secondly, the predetermined and even height of a crown margin does not follow the natural anatomy of the gingival architecture, making the removal of excess cement difficult in the delivery of cemented prostheses [17,18]. Lastly, the color of titanium abutments may lead to a grayish discoloration of peri-implant soft tissue at the cervical level and cause esthetic concerns [18].

In order to solve these issues, customized abutments of different materials have been developed. Besides the morphological advantages of customization, non-metal materials, such as ceramics, zirconia and polymers are esthetically superior due to their tooth resembling color [18]. Abutment material has shown to be able to affect the stability of peri-implant mucosa and crestal bone as well [19]. When selecting the materials for abutments, clear prerequisites are a proven biological compatibility for assuring long-term stability, together with optimal biomechanical and physical properties [20,21].

Materials such as metals, ceramics (alumina and zirconia), and composites are used for the fabrication of individually customized prosthetic abutments [17].

Cast gold individual customized abutments were considered as a state-of-the-art prosthetic solution for a long time; however, recently, their use has been decreasing due to higher pricing and esthetic and biocompatibility issues. In an animal study by Abrahamsson et al., peri-implant soft tissue did not form a sufficient seal with gold abutments; therefore, soft tissue recession and crestal bone loss can be expected.

Apart from gold, dental porcelain is also not considered to be a proper material to establish a reliable soft tissue attachment. The least favorable outcome was found with the use of feldspathic ceramic, as the soft tissue recession and bone loss had the highest extent along this material. Mechanical resistance is also a big issue when dealing with porcelain. Apart from gold, ceramic might also be considered as an alternative material for customized implant abutments. Nevertheless, there is a lack of evidence to support or refute the use of porcelain (including feldspatic) for this specific purpose, but some authors report the use of feldspatic ceramic as a valid coating material for veneers, with an acceptable soft tissues margin adaptation [21].

Alumina implant abutments perform well biologically and esthetically, but they present a greater risk of abutment fracture at the implant–abutment connection during clinical use compared to zirconia [19].

Composite resin abutments are considered to be an alternative and have proven to be as strong as zirconia in in vitro tests published by Magne et al. [22,23].

However, the use of composite resin abutments remains limited due to the concerning reaction of the soft peri-implant tissue to the composite.

Zirconia is a biocompatible material that has optimal esthetic and mechanical properties [17]. Zirconia abutments have been routinely preferred as the abutment of choice especially with increasing esthetic demands in patients with a thin, soft tissue biotype [18]. Some advantages of zirconia include its high mechanical strength due to its unique stress induced transformation toughening mechanism, corrosion resistance, and high loading capacity [15]. Compared to titanium, a zirconia abutment enhances the peri-implant health by reducing inflammation and with less bleeding on probing being present [16].

Even though clinical studies have shown that zirconia abutments indicate very good biological and technical outcomes [19], clinicians still often face the dilemma of choosing between titanium or zirconia abutments. There is so much to keep in mind, such as the biological and esthetic outcome and the mechanical strength between these two implant abutment materials.

Therefore, the objective of this study was to systematically analyze the existing literature to compare the clinical outcomes of titanium and zirconia implant abutments and their modifications. In particular, given the great amount of papers on the topic, the study was designed as a systematic review of systematic reviews [24].

In fact, although several original papers and systematic reviews have been already published on this argument, their outcomes are often inconsistent. Conducting a systematic review of the reviews might help to address this issue in order to highlight evidence from the best quality systematic reviews and bring it together in one single document, that could represent a useful guide for decision-making in clinical practice.

## 2. Materials and Methods

The present systematic review is reported in accordance with the guidelines of the Transparent Reporting of Systematic Reviews and Meta-analyses [24,25].

### 2.1. Focused Question

The purpose of the review was to analyze and compare zirconia and titanium implant abutments and their clinical effect on soft and hard peri-implant tissue.

The focused question was set according to the PICO (population or problem (P), intervention (I), comparison (C), and outcome (O)) strategy as follows:

- Population: healthy patients with at least one dental implant connected to a titanium or zirconia abutment.
- Intervention: titanium or zirconia abutment with or without any surface modification.
- Comparison: titanium or zirconia abutment with or without any surface modification.
- Outcome: mechanical, biological and esthetic outcomes.

The biological outcomes included the pocket probing depth, bleeding on probing, soft tissue recession, marginal bone level and biological complications.

The esthetic outcomes included the pink esthetic score (PES) and white esthetic score (WES) as proposed by Belser et al. [26].

### 2.2. Search Strategy

A MEDLINE/PubMed literature search was performed to find relevant systematic reviews published in English up to and including June 2021. The Cochrane Database of Systematic Reviews and SCOPUS were also searched. The main keywords used in the search were: dental implant(s), abutment(s), titanium, zirconia, combined using AND/OR as Boolean operators.

We hand-searched the contents pages of the most relevant journals in the field. In addition, the search was complemented by manual searches of the reference lists of all the systematic reviews captured.

### 2.3. Inclusion and Exclusion Criteria

The criteria for the study inclusion were as follows:

- Systematic reviews evaluating the analyzed outcomes.

The exclusion criteria were as follows:

- Systematic review of in vitro studies.
- Systematic reviews of animal studies.
- Studies focused on the implant–abutment interface.
- Studies comparing titanium and zirconia implants.

No language restrictions nor restrictions related to the year of publication were imposed. Studies not meeting the inclusion criteria were excluded.

### 2.4. Screening Method

Two reviewers (H.F.C. and P.P.) independently performed the primary search and afterwards, the screening of the titles and abstracts was done manually. The full texts of potentially eligible reviews were obtained and independently assessed by the two reviewers to make a decision and select the studies that met the inclusion criteria. Any discrepancy was resolved through a discussion with a third author (M.M.).

### 2.5. Data Extraction

Data were extracted independently by two reviewers (H.F.C. and P.P.) using an Excel spreadsheet (Microsoft, Redmond, WA, USA) specifically created for this review. The data extracted included: the title, authors, year of publication, type of study (e.g., systematic review or systematic review and metanalysis), focused question of the study clearly reported, abutment materials mentioned, number of studies included, years in which the studies in the systematic reviews were published, total number of patients reported, total number of implants reported, total number of abutments reported (divided into three different groups of materials: titanium, zirconia, and others), a comparison of other abutment modifications, and outcomes. During this process, any discrepancy was resolved through a consensus discussion with a third author (M.M.).

### 2.6. Quality Assessment

The AMSTAR 2 (a measurement tool to assess systematic reviews), as suggested by Shea BJ et al., was used to assess the methodological quality of the systematic reviews included in the present research [27].

## 3. Results

### 3.1. Inclusion and Exclusion of Articles

**Figure 1:**
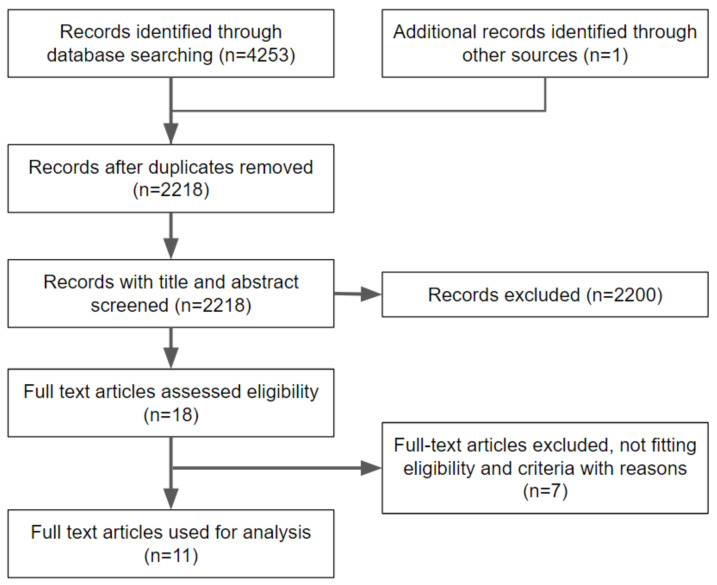
Flow diagram of study selection process.

A flow diagram reporting the screening and selection of studies is presented in Figure 1. The electronic search identified 4253 papers in total, and after a duplicates removal, 2218 articles were screened. After a manual screening of the title and abstract, 2200 studies were excluded, and the full-text of 18 articles was analyzed. Further analysis was performed to make sure that the articles matched the inclusion/exclusion criteria of the present review. Seven additional articles were excluded in this phase. The reasons for exclusion are shown in Table 1.

### 3.2. Description of Selected Articles

Eleven studies published between 2013 and 2020 were selected for the final analysis, and they consisted of nine systematic review and meta-analysis (Linkevicius et al., 2015, Vechiato-Filho et al., 2016, Cai et al., 2018, Sanz-Sánchez et al., 2018, Hu et al., 2019, Cao et al., 2019, and Pitta et al., 2020) and three systematic reviews (Bidra et al., 2013, Naveau et al., 2019, and Gou et al., 2019) as displayed in Table 2. Eight studies reported a comparison between titanium and zirconia abutments, two articles focused on zirconia abutments only and one article reported multiple comparisons related to the following aspects: the abutment material, macroscopic design, surface topography and surface manipulation. Five articles also included a comparison with other abutment materials, such as, gold, alumina, lithium disilicate, hued titanium, and titanium nitride. Articles that focused on titanium abutment modifications did not meet the inclusion criteria. The report on the mechanical, biological and esthetic outcomes is limited to an overall description.

### 3.3. Quality Assessment

A quality assessment of the 11 studies selected is reported in Table 3.

### 3.4. Comparison of Titanium and Zirconia Abutments

There are plenty of case reports and clinical trials comparing different materials for implant abutments. The majority of them discuss titanium and zirconia, as titanium is considered to be the gold standard [17] and zirconia has proven to be biocompatible with peri-implant tissues, to provide a high strength and better esthetic promise compared to titanium, due to its whitish color [26].

### 3.5. Mechanical Outcomes

Five articles (Bidra AS et al., Vechiato-Filho AJ, et al., Sanz-Sánchez I, et al., Naveau A, et al., and Gou M, et al.) discussed the mechanical outcomes of titanium and/or zirconia abutments. There were four mechanical outcomes, which are divided into four different tables reported below: an abutment fracture, screw loosening, abutment screw fracture and veneer failure (Table 4).

### 3.6. Biological Complications

As for the biological outcomes, there were eight studies (Bidra AS, et al., Linkevicius T, et al., Vechiato-Filho AJ, et al., Sanz-Sánchez I, et al., Hu M, et al., Cao Y, et al., Naveau A, et al., and Sanz-Martín I, et al.) that reported outcomes. These outcomes included biological complications, recession, pocket probing depth bleeding on probing (BOP) and plaque index and marginal bone loss (Table 5).

### 3.7. Esthetic Outcomes

Esthetic outcomes (Table 5) were discussed in eight reviews (Bidra AS, et al., Linkevicius T, et al., Cai H, et al., Sanz-Sánchez I, et al., Hu M, et al., Pitta J, et al., Naveau A, et al., and Sanz-Martín I, et al.).

## 4. Discussion

The objective of the present systematic review was to analyze and compare the clinical outcomes of titanium and zirconia implant abutments and their modifications, by searching systematic reviews on this topic. This objective has been studied for many years and numerous studies have been published, from simple case reports to systematic reviews. Although titanium and zirconia are not the only materials that were studied, both materials have shown to have high percentages of success as implant abutment materials [42,43,44]. Clinicians should balance the functional and esthetic performance when making clinical decisions regarding the abutment material selection [1]. In general, the results have reported no significant difference between titanium and zirconia abutments under the mechanical, biological and esthetic aspects.

### 4.1. Mechanical Outcomes

There were four main mechanical outcomes that were discussed in the present systematic review reporting a higher incidence of fracture complication in ceramic abutments compared to titanium ones [19]. The inherent properties of ceramic materials, with a lower resistance to fracture and less flexural strength when compared to metals, may explain these findings [19,38]. The risk of abutment fracture may also be affected by the thickness of the material and the position and angulation of the implant with respect to the final prosthetic restoration [19]. To decrease the risk of an abutment fracture, the wall thickness of zirconia abutments must be maintained above 0.5 mm during manufacturing, while titanium has to be preferred when the thickness is less than 0.5 mm [39]. Manufacturers have also restricted the indications for zirconia abutments to limited angulation. Stock abutments provide maximum angulation of 15 to 20 degrees, while CAD-CAM custom abutments are not recommended for an angulation of over 30 degrees [40]. The abutment fracture rate of zirconia varies from 1.08% to 17.86% depending on age, gender, tooth position, abutment systems, implant systems and implant–abutment connections. Abutment fractures are more likely to happen in young male patients, most probably due to higher occlusal forces. In addition, zirconia abutments in the posterior areas seem to be more susceptible to fractures, compared to placement in the anterior areas [39].

Implant–abutment connection design also plays a huge role in the success of zirconia abutments. A one-piece internal connection showed the highest fracture rate, while external and two-piece internal connections reported a lower fracture rate [39]. Most zirconia abutment fractures occurred within 3 years after loading and several occurred during the initial fitting or subsequent tightening under a controlled torque and the primary fracture location can vary depending on the implant–abutment connection design. In one-piece internal connections, fractures mostly happen in the neck of the implant or below the implant shoulder. The region around the abutment screw head experiences the highest torque and stress concentration and is the most critical region for the stability of ceramic abutments, while the zirconia portion below the implant shoulder is the thinnest and, therefore, cannot resist high torque values. In two-piece internal connection and external connection zirconia abutments, fractures are mostly found above the implant shoulder [39].

An abutment screw fracture was mentioned only in one systematic review and, therefore, seems to be a very rare mechanical complication [42]. Irrespective of the abutment material, abutment screw loosening was found mostly in studies using external hex implants for single implant restorations. Although abutment screw loosening may not be considered a failure, repeated screw loosening can affect the success of implant therapy and patient satisfaction [42]. It has been shown that implant screws are submitted to high concentrations of stress during mastication in the posterior area and this is the most critical area for the stability of ceramic abutments. Nothdurft et al. [45] observed that screw loosening on zirconia abutments may occur due to wear induced by the unavoidable micromovements of the prosthetic component at the implant–abutment interface, which is maximized by the difference in the material properties. Ceramic abutments with titanium bases, or a two-piece Y-TZP abutment, have been proposed as an alternative to solve this problematic wear. Moreover, two-piece abutments have a reduced tendency for complications because they have a higher resistance to micromovements due to ductility of the titanium, which allows some deformation when submitted to unfavorable movements [35]. It was observed that failure in the veneer layer on zirconia abutments was considered as the most frequent problem, especially in all-ceramic restorations. Veneer failure, however, may not lead to implant or prosthetic failure, depending on the extension of the fracture, compromised esthetics or function of the prosthesis. This complication may strongly affect patient comfort and satisfaction as it may increase the treatment time, costs and complexity of maintenance. Veneer failure occurs mostly due to weak bonding between the veneering ceramic and the zirconia infrastructure [35]. When comparing zirconia and titanium abutments, apparently the incidence of a veneer failure on single crowns cemented on titanium abutments was relatively higher compared to zirconia abutments, and this may be associated with the inherent and unavoidable differences in the mechanical properties of ceramic and titanium [35,38].

The type of prostheses and the implant connection are other factors that may influence the outcome [46].

Zirconia abutments presented a better performance in the overdenture group compared to titanium abutments; however, only two original studies reported about implants that were restored with implant-supported overdentures [37].

Although according to several studies the success rates of zirconia and titanium abutments were very similar, further studies need to be performed for two-piece abutments, or hybrid zirconia–titanium abutments. In particular, further clinical research about the mechanical outcomes of this implant abutment design is needed, as some published studies were not specific on how the titanium and zirconia were bonded together as a single unit implant abutment.

### 4.2. Biological Outcomes

As for the biological outcomes, plenty of outcomes were extracted, mostly regarding the material effect towards peri-implant soft tissue. Although zirconia and titanium have both proven to be biocompatible with the surrounding tissue, biological complications were reported in several studies. Buccal fistulas were involved in both screw and cement-retained restorations. In screw-retained restorations, this was only seen in external hexagon implants, and might have been caused by the gap between an ill-fitting abutment and an implant [34]. Increased leakage was reported for zirconia abutments compared to titanium abutments, possibly due to the lower recommended torque values used to tighten the zirconia abutment [47]. In cement-retained restorations, the fistulas were attributed to uncleaned residual cement [16,34]. The design of the abutment might also affect the amount and incidence of cement remnants in peri-implant sulcus. In fact, when the crown margins are located between 1–1.5 mm submucosally, they prevent the complete removal of cement remnants, even when using customized abutments. Resin cement is the most difficult to remove from abutments; therefore, it may be assumed that this complication is dependent on the abutment design and cementation agent, rather than on the abutment material. Hence, supragingival or epigingival margins of abutments are suggested, especially if the implant restorations are to be cemented with resin luting agents [16].

In a systematic review by Sanz-Martín et al. [1], it was reported that there were cases of suppuration without bone loss with no further explanation mentioning the cause of the complication.

The overall survival rate of the two materials shows no significant difference [1,37], but further study with longer follow-ups might be necessary to determine which of the two materials has a better survival rate.

Peri-implant mucosal recession is also considered a biological complication and was reported in several systematic reviews. This complication was predominantly reported in studies using prefabricated titanium abutments. It may be related to the fact that prefabricated abutments provide less optimal support to gingival tissues compared to customized abutments. Regarding this complication, titanium abutments have been reported in more studies because of their longer period of usage, and recession related to titanium abutments can be easily seen and recorded compared to ceramic abutments [34]. Another systematic review stated that soft tissue recession was not influenced by the selection of the abutment material, but by other important factors such as the 3D position of the implant and the presence or absence of attached mucosa [16]. This result was not in accordance with the systematic review of Cao et al. [37] that revealed that zirconia abutments could better reduce the recession in both the junctional epithelium and alveolar ridge compared to titanium abutments, although the reduction was limited [37].

The implant abutment shape design has shown effects on peri-implant soft tissue. In fact, some outcomes are more dependent on the design rather than the material of the abutment. It was reported that a comparison between concave and convex abutments showed that the soft tissue thickness was greater in the concave group [39]. The use of the concave transmucosal profiles for implant components allowed for more predictable soft tissue stability in esthetic areas. In a study by Rompen et al. [48], concave abutments of both titanium and zirconia material were used for single crown restorations, and their results showed that 87% of the sites showed facial soft tissue stability or a vertical gain, and recession in the remaining 13% was below 0.5 mm [34].

A difference was found for mucosal inflammation or bleeding on probing (BOP) when comparing abutment materials. Some systematic reviews found that mucosal inflammation was greater next to titanium abutments compared to zirconia ones [33,37,39], while the macroscopic design, surface topography or surface manipulation did not have a significant influence on this outcome [39]. Lower BOP values may be caused by less plaque retention in zirconia abutments compared to titanium ones due to the surface properties of the material [34,35,38,39]. It must be added that it is more and more common to treat patients with anticoagulant therapy [49].

This finding is also supported by an in vivo study where the bacterial colonization was compared between zirconia and titanium discs attached to a removable dental prosthesis [16,39]. Ceramic abutments are easier to polish than metal abutments, which can greatly reduce the bacterial colonization and adhesion [38]. They are also resistant to corrosion which allows for the better growth of epithelial cells and inhibits bacterial adhesion. Plaque accumulation can also be affected by several other factors besides implant abutment modifications including a cement excess, and a misfit between the prosthesis and the implant platform caused by screw loosening or ill-fitting prosthetic components [35]. Soft tissue attachment also plays a role in the degree of inflammation. In an in vitro study [42], zirconia exhibited a higher degree of fibroblast proliferation when compared to titanium. This result, however, does not translate to the differential histological outcomes in experimental studies comparing abutments made of zirconia, titanium and gold alloy. A similar soft tissue dimension was reported for titanium and zirconia abutments, while in the gold alloy abutments there was an apical shift of the epithelium barrier followed by a marginal bone loss [34,39]. It was reported that healing at zirconia and titanium abutments allowed the formation of a mucosal attachment that included an epithelial and a connective tissue portion that were about 2 mm and 1–1.5 mm high, respectively, which demonstrates that the abutments made of titanium and zirconia promoted the proper conditions for soft tissue healing [14].

Other than the different materials, the surface properties and in particular the roughness may have effects on soft tissue attachment [31]. Some histological studies on animals and humans have demonstrated that moderately rough surfaces can be beneficial for soft tissue integration, but this conclusion cannot be drawn as there is insufficient investigation on this hypothesis [31]. No significant difference was found for the periodontal probing depth between zirconia and titanium implant abutment material; however, in some studies, the cementation margin was placed subgingivally, which could be a setback in the study design. Clearly, if the restoration margin extended deeper subgingivally, the peri-implant tissues at the gingival parameters would contact the restoration material instead of the abutment material. This would influence the periodontal probing depth, accumulation of plaque and other biological parameters [17].

Titanium and zirconia abutments did not seem to influence marginal bone loss. Although zirconia abutments seem to have less marginal bone loss compared to titanium abutments, there was no significant difference between the two materials [20,38]; however, when changes in the marginal bone loss were assessed over time, a significant loss occurred for both materials except for titanium nitride. The magnitude of this loss, with a mean follow-up of 30 months, has limited clinical significance as it is smaller than the mean error of repeated radiographic measurements [20]. Surface decontamination on implant abutments seems to be an important factor affecting the peri-implant bone levels. A greater amount of bone loss was reported for steamed titanium abutments compared to plasma argon titanium abutments. It is known that the strong affinity of titanium to proteins and amino acids makes the complete cleaning of its surface rather difficult, but plasma argon cleaning has been shown to effectively decontaminate titanium surfaces in vitro. In human histological studies, it has been reported that plasma argon may promote cell adhesion and positively influence collagen fiber orientation [39].

The present systematic review of systematic reviews presents some limitations as some of the systematic reviews that were included faced their own difficulties in collecting data with less heterogeneity and bias in the studies included. The lack of differences for some clinical outcomes analyzed may also be due to the questioned reliability of periodontal parameters to assess peri-implant health. Different studies also use different methods to measure those parameters that may affect the overall results of the investigation. It has been shown that factors such as gender, implant position, or age can influence the peri-implant health parameters. Moreover, excessive probing forces may induce false-positive BOP readings and the insertion of a periodontal probe in cases with overhanging restorations may result in lower periodontal probing depth values, increasing the trauma towards the peri-implant soft tissue and leading to a false-positive BOP [39].

### 4.3. Esthetic Outcomes

Another important factor to be considered for the abutment selection is its possible impact on the esthetic outcome of the implant–supported final restoration. The systematic reviews that were included had selected studies with many different esthetic indices used. Studies using spectrophotometry have shown better esthetic results with zirconia abutments compared to titanium abutments, mainly due to the color appearance of peri-implant soft tissue [20]. The blue-grayish shimmering effect of titanium abutments, especially in the case of thin peri-implant mucosal tissues, can compromise the esthetic result [34]. Hu, et al. found no difference in the discoloration of soft tissues with different abutment materials [38]. Peri-implant mucosal thickness is also of importance to achieve satisfactory results, as it has been shown that the abutment material determines minimal color changes in thicker tissue, generally more than 3 mm [17,20,41]. It is recommended to use titanium abutments when the tissue thickness is at least 3 mm, while for zirconia implant abutments, a 2 mm of soft tissue thickness would be enough [41]. Hence, the use of zirconia abutments can be esthetically appreciated only in the case of thin tissue biotype [17]. Additionally, titanium abutments with modified surfaces, such as anodized ones, seem to have more discoloration compared to zirconia [40,41]. Anodized pink titanium abutments present a “grayish” color as they are all-metallic while ceramics present a more “whitish” color [41]. The pink veneering of zirconia seems to be more promising according to spectrophotometric measurement without a significant biological or technical alteration; however, further study is needed [40]. When different abutment materials were compared to natural teeth, both titanium and zirconia abutments induced visible discoloration when assessed using a spectrophotometer in the peri-implant soft tissue [34,41]. It appears that studies using spectrophotometric analysis showed a higher sensitivity to detect peri-implant mucosal discoloration, whereas studies using subjective or objective scoring criteria reported a minimal difference in the esthetic outcomes and patient satisfaction [34]. According to Linkevicius et al. [17], the pink esthetic score (PES) index should be used for the evaluation of the final esthetic outcome because it reflects the soft tissue condition better. The PES was originally proposed by Furhauser et al. [50] and it is composed of five factors, namely, the mesial papilla, distal papilla, curvature of the facial mucosa, level of facial mucosa, and root convexity or soft tissue color and texture at the facial aspect of the implant site. For example, the PES score around zirconia abutments was significantly higher compared to titanium abutments at a 2 year follow-up [17].

There were limitations in the esthetic assessments as well because different systematic reviews included different inclusion and exclusion criteria. In those systematic reviews, different indices were used, hence, the outcome can be greatly influenced when different measurements are taken into consideration.

Additionally, some studies had taken measurements before the cementation of a prosthesis or crown, while others assessed the outcome without prosthesis.

Future research should focus on improving the study methodology in this field of implant dentistry. Although research on implant abutment materials and their modifications have been published for many years, many clinicians still have difficulty in deciding on the abutment material and design. The possible variables in the choice of the implant abutment seems to be limitless, including the material, macroscopic design, surface topography, surface treatment and even the modification of color by veneering in zirconia abutments and anodizing in titanium ones.

Other than that, implant abutment can also be influenced by how a prosthesis is placed, namely, cemented or screw retained. There is a wide variety of factors that play a role in peri-implant soft and hard tissue health. Consequently, clinical trials with strict methodological designs can be beneficial for future systematic reviews, with the aim of supporting clinical choices and leading to clear guidelines regarding the selection of the most appropriate abutment materials and their modifications in each specific clinical case, in order to maximize the implant rehabilitation success and patient satisfaction.

## 5. Conclusions

It can be concluded that:

1. Titanium has proven to be mechanically superior compared to zirconia, although the difference in the incidence of mechanical failures was not significant in the majority of the studies. In the posterior area and where occlusal forces are stronger, a titanium implant abutment is the better option.
2. There is no significant difference in the biological complications, marginal bone loss and periodontal probing depth between zirconia and titanium abutments, although titanium abutments showed a tendency to greater plaque accumulation compared to zirconia ones.
3. BOP is slightly greater next to titanium abutments compared to zirconia ones, but it is not influenced by the macroscopic design, surface topography or surface manipulation.
4. The macroscopic design of implant abutment seems to influence the soft tissue thickness only, with concave abutments allowing more soft tissue gain for both zirconia and titanium implant abutments. The position of the prosthetic margin also plays a big role especially in the case of cement-retained prostheses.
5. Surface roughness or topography may play a role in soft tissue attachment; however, further research is needed to confirm this hypothesis.
6. Marginal bone loss was not significantly influenced by the abutment material, but by the surface decontamination of titanium implant abutments. Plasma argon titanium abutments show less bone loss compared to steamed titanium abutments.
7. Regarding the esthetic outcome, any implant abutment material can be used when the soft tissue thickness is sufficient (more than 3 mm). In cases with limited soft tissue thickness, especially in the esthetic area, zirconia abutments show better esthetic results. Further research is needed to evaluate modifications that can be made to both materials, such as anodization or veneering of the abutment.

## Figures and Tables

**Table 1:** The table shows reasons for exclusion of 7 articles after reading the full-text.

	Excluded Articles	Year	Reason of Exclusion
1	Canullo L, Pesce P, Patini R, Antonacci D, Tommasato G. [28]	2020	Outcome cannot be retrieved
2	Al Rezk F, Trimpou G, Lauer HC, Weigl P, Krockow N. [29]	2018	Includes animal and in vitro studies
3	Linkevicius T, Apse P. [30]	2008	Includes animal studies
4	Pesce P, Menini M, Tommasato G, Patini R, Cannullo L [31]	2019	Discusses healing abutment modification
5	Canullo L, Menini M, Santori G, Rakic M, Sculean A, Pesce P. [2]	2019	Discusses healing abutment modification
6	Yu SB, Song BG, Cheon KJ, Kim JW, Kim YH, Yang BE. [32]	2018	Narrative review
7	de Medeiros RA, Vechiato-Filho AJ, et al. [33]	2013	Evidence based narrative literature review

**Table 2:** Main characteristics of the articles included in the present review.

	Authors	Year of Publication	Design of the Studies Included	Focused Question	Databases Searched	n. of Studies Included	n. of Patients	n. of Implants	Abutment Materials Investigated	Outcomes Investigated
1	Bidra AS, et al. [34]	2013	RCT, prospective, retrospective, and cross sectional studies	Evaluate clinical outcomes including survival outcomes, mechanical outcomes, and biological and esthetic outcomes of implant abutments used exclusively in the maxillary and mandibular anterior regions.	Pubmed/Medline	27 studies	NA	NA (implants abutments only in the anterior)	Titanium, cast metal alloy, alumina, zirconia and zirconia with titanium base abutments	Survival, mechanical, biological and esthetic outcomes
2	Linkevicius T, et al. [17]	2015	Clinical studies	To define the effect of zirconia and titanium as abutment materials on soft peri-implant tissues. The topic was divided into 2 parts: (a) biology and (b) esthetics.	Pubmed/Medline	11 studies	389 patients	512 implants (280 titanium abutments and 232 zirconia abutments)	Titanium and zirconia abutments	Biological and esthetic outcomes
3	Vechiato-Filho AJ, et al. [35]	2016	RCT and prospective studies	Are zirconia implant abutments safe and predictable in posterior areas?	Pubmed/Medline and Cochrane Library	11 studies	353 patients	NA	Titanium and zirconia abutments	Mechanical or biological complications
4	Sanz-Sánchez I, et al. [20]	2018	RCT, CCT and case series studies	Which is the effect of the abutment material on the stability and health of the peri-implant tissues?	Pubmed/Medline and Cochrane Central Register of Controlled Trials	29 studies	NA	NA	Titanium, zirconia, alumina, Li Dis, gold,	Biological, mechanical and esthetic outcome
5	Sanz-Martín I, et al. [1]	2018	RCT	Which is the effect of modifying the abutment characteristics for maintaining peri-implant soft tissue health?	Pubmed and Cochrane Central Register of Controlled Trials	13 studies	NA	889 implants	Titanium, alumina, zirconia, and ceramic	Biological outcome (peri-implant soft tissue health)
6	Cai H, et al. [36]	2018	RCT, prospective and retrospective studies	How do zirconia and other abutments with different tints affect the color of peri-implant soft tissue?	PubMed, EMBASE, Cochrane Database of Systematic Reviews (CDSR), and Cochrane Central Register of Controlled Trials (CENTRAL)	8 studies	NA	365 implants (128 titanium abutments, 141 zirconia abutments, 96 gold abutments (not reported in all included studies))	Titanium, zirconia, and golden abutments	Esthetic outcome (quantitative discoloration assessment)
7	Cao Y, et al. [37]	2019	RCT, CCT, and long-term observational studies	In patients treated with titanium implants with zirconia abutments, what percentage of implants can survive, and what is the effect of zirconia abutments on the marginal bone loss (MBL) and pocket probing depth (PPD), compared with all-titanium implants?	Cochrane Central Register of Controlled Trials (CENTRAL), MEDLINE via OVID, EMBASE, and Chinese Biomedical Literature Database	18 studies (10 studies included in part 1, 12 studies included in part 2)	Part 1: NA Part 2: 332 patients	Part I: 353 implants Part 2: 427 implants	Titanium and zirconia abutments	Biological outcomes
8	Hu M, et al. [38]	2019	RCT, CCT, prospective and retrospective studies	How do abutment materials influence the survival rate of the abutment, the marginal bone loss and the peri-implant soft tissue discoloration in implant-supported single crowns?	Medline, EMBASE, Web of Science, CENTRAL (Cochrane Library), CNKI (China National Knowledge Infrastructure), and the Chinese Biomedical Literature Database	23 studies	NA	1006 implants (403 titanium abutments, 35 alumina abutments, 447 zirconia abutments, 121 golden abutments)	Titanium, zirconia, alumina and golden abutments	Biological and esthetic outcome
9	Gou M, et al. [39]	2019	RCT, case reports, prospective and retrospective studies.	To determine the characteristics, causes, managements, and preventive measures with respect to zirconia abutment fracture.	Medline, Embase, and Cochrane library	15 studies	NA	1528 implants	Zirconia abutments	Mechanical outcomes
10	Naveau A, et al. [40]	2019	RCT, prospective and retrospective studies.	In patients requiring a single, anterior implant, what are zirconia abutments’ survival, mechanical, and esthetic outcomes?	Pubmed/Medline	20 studies	NA	NA	Zirconia abutments	Mechanical and esthetic outcomes
11	Pitta J, et al. [41]	2020	RCT	(1) Do ceramic abutments exhibit differences in peri-implant soft tissue color when compared to metallic abutments in single-unit implant supported reconstructions? (2) Does the soft tissue thickness have an effect on the peri-implant soft tissue color differences when metallic or ceramic abutments are used for single-unit implant-supported reconstructions?	Pubmed/Medline	6 studies	265 patients	NA	Titanium, zirconia and gold alloy abutments	Esthetic outcomes

**Table 3:** Quality evaluation.

Criteria	Bidra AS et al. [34]	Linkevicius T et al. [17]	Vechiato-Filho AJ et al. [35]	Sanz-Sánchez I et al. [20]	Sanz-Martín I et al. [1]	Cai H et al. [36]	Cao Y et al. [37]	Hu M et al. [38]	Gou M et al. [39]	Naveau A et al. [40]	Pitta J et al. [41]
1. Research question and inclusion criteria PICO											
2. Protocol registered before commencement of the review											
3. Explanation of selection of drawings from the included studies											
4. Adequacy of the literature search											
5. Duplicate study selection											
6. Duplicate data extraction											
7. List and justification of excluded studies											
8. Studies included described in detail											
9. Risk of bias from individual studies being included in the review											
10. Sources of financing of included studies reported in review											
11. Appropriateness of meta-analytical methods											
12. If meta-analysis: bias risk of included studies taken into account											
13. Risk of bias taken into account in the interpretation and discussion											
14. Satisfactory explanation for any heterogeneity											
15. Assessment of presence and likely impact of publication bias											
16. Conflicts of interest											

Legend: Criterion identified in the text 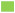; criterion partially identified in the text 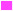; unidentified criteria in the text 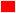; not applicable 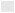.

**Table 4:** Mechanical outcomes reported in included systematic reviews.

Authors	Abutment Fracture	Screw Loosening	Abutment Screw Fracture	Veneer Failure
Bidra AS, et al. [34]	11 studies reported fractures of ceramic abutments, 8 on alumina abutments and 3 on zirconia abutments. No fractures were found in titanium or cast metal abutments.	Abutment screw loosening was reported as primary mechanical complication, although screw loosening is a well-recognized complication for external hex implants which was used in the majority of these studies.	Only one study reported an abutment screw fracture, making it appear to be a rare complication for anterior abutments.	-
Linkevicius T, et al. [17]	-	-	-	-
Vechiato-Filho AJ, et al. [33]		Screw loosening was reported for both materials.		In the posterior area, the risk ratio (RR) showed that zirconia abutments were about 0.52 times more susceptible to veneer failure than titanium abutments. Veneer failure mostly occurs in the cusp tips and polishing was considered adequate to overcome the problem.
Sanz-Sánchez I, et al. [20]	Abutment fracture was reported. There was a higher, but non-significant, incidence of complications for ceramic when compared to titanium abutments.			Veneer chipping was reported.
Sanz-Martín I, et al. [1]				
Cai H, et al. [36]				
Cao Y, et al. [37]				
Hu M, et al. [38]				
Gou M, et al. [39]	Posterior teeth seemed more susceptible to zirconia abutment fracture. Fewer fractures were reported for two-piece internal connection zirconia abutments compared to external connection zirconia abutments and one-piece internal connection zirconia abutments. One-piece internal connection zirconia abutments had the highest fracture rates. Higher fracture rates were reported for abutments with platform switching compared to standard platforms.			
Naveau A, et al. [40]	Considered as a major mechanical complication, varying from 1.2% to 8%, fractures were found in screw access holes with thin walls for abutments with an external connection, while for internal connections it was found in the implant necks. Implant diameter did not seem to have any influence on the fracture rate and no specific time frame could be defined.	Considered as a minor mechanical complication. The highest rate of screw loosening found was 6% in one study.		
Pitta J, et al. [41]				

**Table 5:** Biological complications and esthetic result as reported in included systematic reviews.

Authors	Biological Complications	Recession	Pocket Probing Depth	Bleeding on Probing and Plaque Accumulation	Marginal Bone Loss	Esthetic Outcome
Bidra AS, et al. [34]	As for biological outcomes, fistulas were found to be the most common complication, both in screw-retained and cement-retained restorations.	Peri-implant mucosal recession was reported predominantly in studies using prefabricated titanium abutments. Concave-shaped abutments showed better soft tissue stability, minimized soft tissue recession, and even a gain in soft tissue height as reported in two studies.	-	-	-	In studies using spectrophotometric analysis, zirconia abutments showed less peri-implant mucosal discoloration compared with metal abutments.
Linkevicius T, et al. [17]	12 out of 145 zirconia abutments experienced biological complications including a buccal marginal fistula, swelling, pain, suppuration, suppuration at probing, and a pocket probing depth of more than 5 mm. 5 titanium abutments out of 110 had biological complications, varied from a fistula, mucositis, suppuration at probing, a probing depth of more than 5 mm and a failure of the implant. The percentage of biological complications in titanium abutments was lower compared to zirconia abutments	In one study, there was slightly higher recession found in titanium abutments compared to zirconia, although there was no significant difference during a 5 year follow-up. In another study it was reported that stock titanium and zirconia abutments showed similar amounts of soft tissue recession while there was less recession in CAD/CAM zirconia abutments and an increase in soft tissue for CAD/CAM titanium abutments; although, the differences between the 4 groups were also not significant. Another study reported different results as the soft tissue recession was measured in two sites, the mesial and distal. In both titanium and zirconia abutments, there was soft tissue gain in the mesial site while soft tissue recession occurred in the distal site.	Pocket probing depth was reported to be slightly higher in titanium abutment but there was no significant difference between both materials.	Bleeding on probing was found to be slightly higher in zirconia abutments compared to titanium abutments, but the difference was not significant.	Marginal bone loss results were very similar between the two materials.	Soft tissue color around zirconia abutments showed a better color match to natural teeth compared to titanium abutments but no statistical differences was observed after several years of follow-up. Indexes such as the Copenhagen Index Score (CIS), Implant Crown Aesthetic Index (ICAI) and Papilla Index also showed no significant difference between both abutment materials, but zirconia had slightly higher scores in all indexes when compared to titanium. The Pink Esthetic Score (PES) was measured in one study during 12 months and 24 months of follow-up. The score was higher for zirconia, showing a significant difference between the two materials for both follow-up periods.
Vechiato-Filho AJ, et al. [35]	There was no significant difference in biological complications: mean bone loss for zirconia was 0.38 ± 0.87 mm and 0.2 ± 0.13 mm for titanium abutments; success rates were 99.3% for zirconia abutments and 99.57% for titanium abutments in the posterior area.					
Sanz-Sánchez I, et al. [20]	Overall incidence of biological complications was low.		Abutment material had no influence on probing depth.	Titanium showed a greater increase in BOP and greater plaque accumulation when compared to zirconia.	No significant difference was found between titanium and zirconia. When marginal bone loss was assessed over time, a significant loss occurred in all materials except for titanium nitride.	No differences in the esthetic outcome could be attributed to the abutment material.
Sanz-Martín I, et al. [1]	Suppuration without bone loss was reported in one study for both titanium and zirconia abutments during the one-year follow-up.	Crown length of the implant restoration (CLI) was reported to increase in titanium abutments in one study. Abutments cleaned with plasma argon also showed higher recession compared to conventional/steam cleaning methods.		There was a significant increase in mucosal inflammation (BOP) for titanium abutments when compared to zirconia abutments; however, surface topography or manipulation did not have significant influence on soft tissue inflammation; trend for higher plaque accumulation for titanium abutments compared to zirconia abutments.	No significant difference was found when comparing abutment material and macroscopic design. The difference was significant when comparing surface manipulation, with greater bone loss reported for steamed titanium abutments compared to plasma argon titanium abutments.	Using a Visual Analog Scale (VAS), patients were equally satisfied regarding the esthetic outcome when comparing zirconia and titanium abutments.
Cai H, et al. [36]						Soft-tissue discoloration was significantly lower around zirconia abutments than around titanium or golden abutments.
Cao Y, et al. [37]	Survival rate of titanium implants with zirconia abutments appeared to be lower than those with titanium abutments in the long term.		Results favored implants with zirconia abutments.		Results favored implants with zirconia abutments.	
Hu M, et al. [38]	Survival rate of titanium abutments and zirconia abutments were similar.				Zirconia abutment is better than gold or titanium abutment in terms of maintaining marginal bone.	There seemed to be no difference between zirconia and titanium abutments in discoloration of peri-implant soft tissue.
Gou M, et al. [39]						
Naveau A, et al. [40]					Some studies reported that marginal bone loss was less with zirconia abutments compared to metal abutments.	Zirconia abutments provided better matching and integration of the color and surface of soft tissues than titanium abutments. They were particularly indicated in patients with thin peri-implant mucosa, because thick tissues are necessary to mask the grey color of the titanium abutment.
Pitta J, et al. [41]						No significant difference was found between titanium and zirconia abutments, with limited information on the correlation between soft tissue thickness and ΔE values.

## Data Availability

No new data were created or analyzed in this study. Data sharing is not applicable to this article.
